# Scintigraphic evaluation of salivary gland function in thyroid cancer patients after radioiodine remnant ablation

**DOI:** 10.1111/eos.12689

**Published:** 2020-04-02

**Authors:** Eva Krčálová, Jiří Horáček, Filip Gabalec, Pavel Žák, Jiří Doležal

**Affiliations:** ^1^ Nuclear Medicine Department University Hospital Hradec Králové Hradec Králové Czech Republic; ^2^ Academic Department of Internal Medicine Faculty of Medicine in Hradec Králové Charles University Hradec Králové Czech Republic; ^3^ 4th Department of Internal Medicine University Hospital Hradec Králové Hradec Králové Czech Republic

**Keywords:** ^99m^Tc – pertechnetate, radioiodine remnant ablation, salivary gland dynamic scintigraphy, thyroid cancer, xerostomia

## Abstract

Radioiodine (^131^I, RAI) has traditionally been used in thyroid cancer treatment but its benefit should be balanced against possible risks. Among them, salivary gland dysfunction has often been discussed, although the reported data have been inconsistent. The aim of our prospective study was to evaluate salivary gland function in 31 thyroidectomised patients (6 men, 25 women; median age 52 yr) before and 4–6 months after RAI remnant ablation (RRA), using activity of 3.7 GBq ^131^I‐NaI. Salivary gland uptake and excretion fractions were quantitatively assessed with ^99m^Tc – pertechnetate salivary gland scintigraphy. Pre‐ and post‐treatment values were compared using Wilcoxon signed rank test. No statistically significant difference in the pre‐ and post‐treatment values was observed in parotid or submandibular glands uptake, or in the parotid or submandibular excretion fractions. The calculated power for minimum relevant difference of 25% with the sample size of 31 ranged between 86% and 96% for the individual variables, making our negative results reasonably reliable. The results suggest that RRA with the most commonly used activity of 3.7 GBq has no important impact on salivary gland function. Therefore, the concerns about putative salivary gland functional deterioration following RRA are probably unjustified.

Thyroid cancer incidence is steadily growing worldwide, with low‐risk tumours prevailing (1, 2). Radioiodine (^131^I, RAI) has been used for thyroid remnant ablation, as an effective part of differentiated thyroid cancer therapy (3). Recently, the 2015 American Thyroid Association Management Guidelines for Adult Patients with Thyroid Nodules and Differentiated Thyroid Cancer recommended a more personalised approach, and in low‐risk patients the indication for thyroid remnant ablation should reflect the expected benefit together with possible risks of RAI (4).

Among the risks, salivary gland radiation damage has often been discussed (5). Similar to the thyroid, salivary glands express sodium/iodide symporter (NIS), which may make them susceptible to damage from RAI accumulation, potentially leading to salivary gland hypofunction and clinically pronounced xerostomia (6, 7). As saliva has important local effects (mediating taste sensations, preventing dysbiosis of oral microflora, remineralising enamel) as well as systemic ones (proper digestion of food and a whole range of immune and defensive processes) (8, 9), hyposialia may be a serious problem.

Numerous studies have reported sialadenitis and xerostomia as the most frequent minor side effects of RAI, significantly affecting quality of life due to an increase in caries rate, taste alteration or problems with chewing and swallowing (10, 11, 12, 13). However, these studies were retrospective and based on subjective data from hospital records, questionnaires or interviews, and their methodology often makes their conclusions less reliable (14). In particular, the long intervals between thyroid remnant ablation and patients interviewing (ranging from 1 up to 21 yr) in the frequently cited study by alexander *et al.* (10) are rather concerning.

In contrast, a recent dosimetric study using ^124^I NaI PET/CT shows that the average organ dose per commonly administered RAI activity is about one order of magnitude lower than the external beam radiotherapy dose necessary to induce salivary gland radiation injury (15).

Both sialometry and dynamic salivary gland scintigraphy represent well established and validated options for quantitative salivary gland functional evaluation (16, 17). Sialometry directly measures the volume of total and glandular saliva (using dedicated intraoral devices) under basal conditions and after stimulation. Additionally, biochemical analysis of collected saliva provides valuable information about changes in saliva composition.

On the other hand, non‐invasive salivary gland scintigraphy, using the physiological tracer ^99m^Tc‐pertechnetate (taken up by both the ductal NIS and acinar Na^+^/K^+^/Cl^‐^ co‐transport system), allows the calculation of both the glandular uptake (reflecting parenchymal integrity) and excretion fraction (reflecting the ability to release an adequate saliva volume after a gustatory stimulus) of each salivary gland simultaneously, without intraoral manipulations (16, 18, 19).

In several carefully conducted studies (17, 20, 21, 22, 23, 24), salivary gland scintigraphy or sialometry has been used for quantitative assessment of possible salivary gland dysfunction after RAI administration, sometimes reporting dose‐dependent functional changes, predominantly in parotid glands. In these reports, widely varying and usually higher activities (ranging from 3.7 to 33.9 GBq) have been used.

For thyroid remnant ablation, however, most differentiated thyroid cancer patients are treated with 3.7 GBq (100 mCi), and in very low‐risk patients even lower activity (1.1 GBq) has been advocated (25, 26). Surprisingly, objective sialometric or scintigraphic data on the most commonly used RAI activity of 3.7 GBq are scarce.

Therefore, the purpose of this prospective study was to assess objectively, using salivary gland scintigraphy, whether RAI activity of 3.7 GBq can cause important functional changes in salivary glands. Additionally, we evaluate patients’ hyposialia symptoms using the validated Radiation Therapy Oncology Group modified University of Washington Head and Neck Symptom Scale questionnaire (RM‐UWHNSS) (27).

## Material and methods

### Patients

We performed salivary gland scintigraphy on 31 differentiated thyroid cancer thyroidectomised patients before thyroid remnant ablation (Table 1). None of them had a history of salivary gland disease, systemic autoimmune disease, or head and neck external beam radiotherapy. Only 11 out of 31 patients were taking medications which might interfere with salivation and only one of them used antipsychotics with risk of salivary gland side effects higher than 10%. The details are given in Table 2. Moreover, none of the patients complained of dry mouth symptoms and their medication remained unchanged during the follow‐up. All patients had metallic dental restorations.

**Table 1:** Patient characteristics

Variable	Value
All patients, *n*	31
Gender, *n*	
Male	6
Female	25
Age (yr)	
Median (IQR)	52 (42–60)
Histology, *n*	
PTC	28
FTC	2
Oncocytic	1
Risk stratification according to 2015 ATA guidelines, *n*	
Low‐risk	19
Intermediate‐risk	12
High‐risk	0
TSH before RAI (mIU l^−1^)	
Median (IQR)	89.8 (65.7–112.0)

ATA, American Thyroid Association; FTC, follicular thyroid cancer; IQR, interquartile range; PTC, papillary thyroid cancer.

**Table 2:** Patient medication overwiev

Risk of salivary glands adverse effects* (%)	Number of patients (*n*)	Medication used
0	20	None
0.1–1	6	Amlodipine, perindopril, levocetirizine
1–10	4	Solifenacin, clonazepam, rilmenidine
>10	1	Chlorprothixene

* According to the individual summary of product characteristics.

The patients were then treated with RAI (orally administered activity of 3.7 GBq). For all patients, this was their first RAI treatment. They were encouraged to drink 2.5–3 l of liquids per day. Sialagogues (sour candies, lemon juice, etc.) were not recommended in the first few hours after RAI administration due to the reported risk of salivary gland hyperaemia and higher RAI uptake (28, 29, 30).

Patients were examined again about 4–6 months (median 4.6 months) after thyroid remnant ablation, and a paired comparison was made with the results obtained before thyroid remnant ablation. This interval is long enough to reveal RAI–induced damage to the salivary gland ductal cells (with turnover 60–120 d) (31).

All subjects have given their written informed consent. Our research has been conducted in full accordance with ethical principles, including the World Medical Association Declaration of Helsinki and has been independently reviewed and approved by the University Hospital Hradec Králové Ethical Committee.

### Dynamic salivary gland scintigraphy

Salivary gland functional parameters were assessed according to the protocol validated by klutman *et al.* (16). The imaging was performed using a single‐headed gamma camera MB 9200 (Gamma Müvek, Budapest, Hungary) equipped with a parallel hole, a low energy high resolution collimator. Dynamic scan acquisition (matrix 128 × 128 × 16, 90 frames, each per 20 s, photopeak centred at 140 keV with 10% symmetric window) started immediately after 185 MBq ^99m^Tc‐pertechnetate intravenous administration. Patients laid supine with head stabilised in slightly recline position using a U‐shaped support during the data acquisition. As a gustatory stimulus, lemon juice (3 ml, Realemon; Meroso Foods, Ramsdonk, Belgium) was used; it was administered *per os* 20 min after radiotracer injection.

**Fig. 1:**
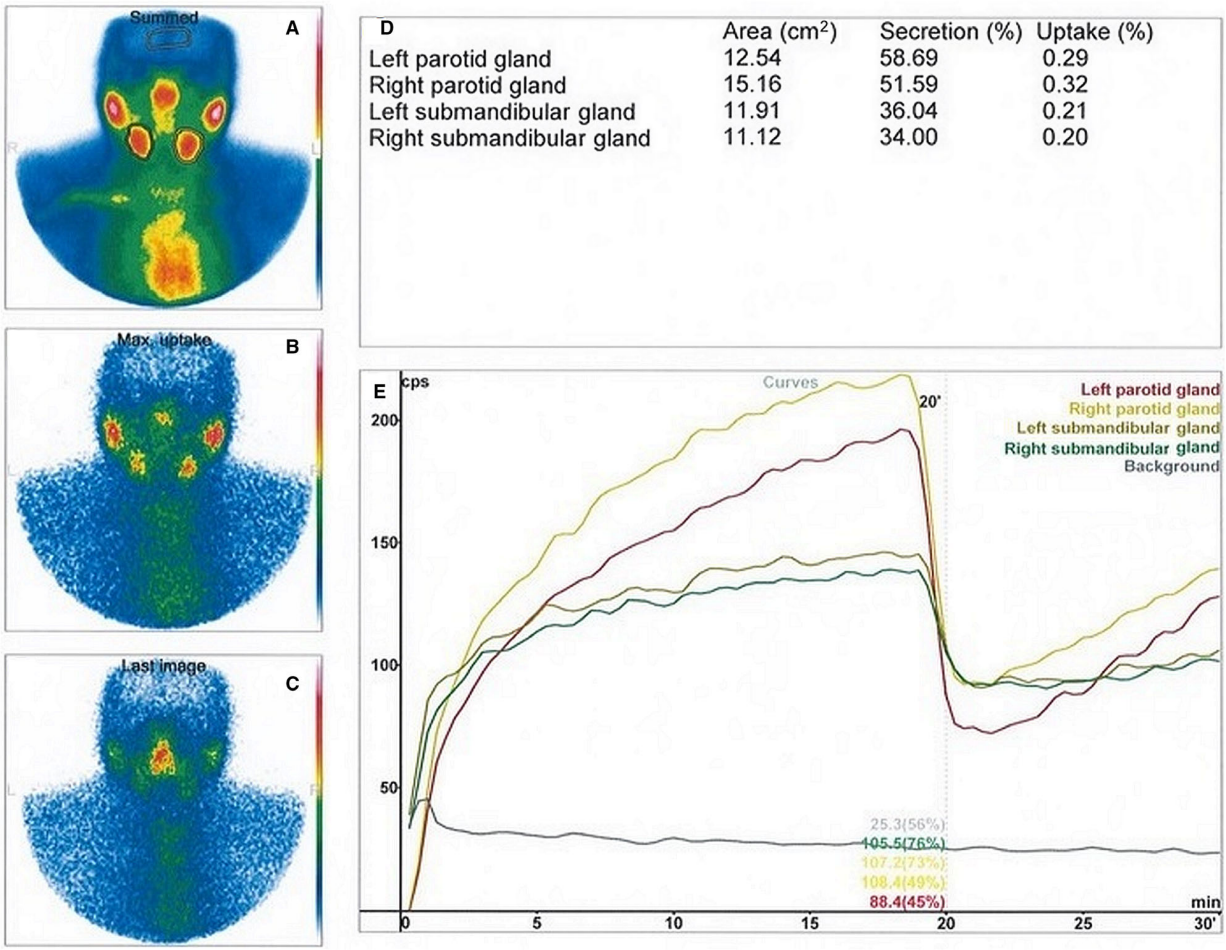
Physiological uptake and excretion of ^99m^Tc‐pertechnetate in parotid and submandibular salivary glands detected by dynamic salivary gland scintigraphy. Summed image (A) obtained from all frames clearly depicts salivary glands, submandibular glands, ROIs and ROI for background correction placed over the brain. Picture (B) shows maximal radiotracer uptake in the salivary glands and (C) shows substantially decreased radiotracer activity in salivary glands 10 min after salivary glands gustatory stimulation. Values in (D) represent calculated uptake and excretion fractions and (E) shows time‐activity curves for each salivary gland.

Data were processed using dedicated software (interview xp; Mediso, Budapest, Hungary). Regions of interest (ROI) were drawn over each salivary gland and one for background correction was created over the brain. The time‐activity curves of each ROI were subsequently generated (Fig. 1).

Salivary gland uptake was calculated as the background‐subtracted percentage of total ^99m^Tc–pertechnetate administered activity measured in the 18^th^ min after the injection. Salivary gland excretion fraction was calculated as the difference between pre‐stimulation and post‐stimulation uptake expressed as the percentage of the pre‐stimulation one (16).

In each patient, the uptake and excretion fraction for left and right parotid glands and for left and right submandibular glands were pooled (due to correction of any tilt), and the average values for each gland were further evaluated.

### Symptom evaluation

Hyposialia symptoms were assessed in all 31 patients simultaneously with baseline and follow‐up salivary gland scintigraphy using the RM‐UWHNSS questionnaire. The questionnaire consisted of 15 questions grouped into 10 independent domains (27). The employment domain was excluded due to its irrelevancy. The scale for symptoms evaluation ranging from 20 to 100 points (with higher score indicating more pronounced symptoms) was used for RM‐UWHNSS questionnaire evaluation according to the dedicated methodology (32). Change scores were calculated by subtracting baseline from follow‐up scores (thus, a positive change score indicated worsening pain), and anything higher than 5 points difference was considered to be meaningful (32).

### Analysis of RAI uptake in dental restorations

Post‐therapeutic planar whole‐body scans, performed 140 h after RAI ingestion, were used for visual assessment of RAI uptake in dental restorations. Uptake intensity was evaluated using a three‐point scoring system (0 ‐ background‐level uptake, 1 ‐ mild uptake, 2 ‐ intense uptake). The RAI uptake score was calculated as a sum of the left‐ and right‐side scores. Single Photon Emission Tomography/Low Dose CT (SPECT/CT) was used for confirmation of RAI uptake in metallic dental restorations.

### Statistical analysis

For statistical analysis, sigmastat software package (Jandel, San Rafael, CA, USA), version 3.1, was used. As the data were mostly non‐normally distributed, their summary values were expressed as medians and interquartile ranges (IQR), and comparisons of parotid and submandibular glands average uptake and excretion fractions before and after RRA were performed using Wilcoxon signed rank test. A *P*‐value of <0.05 was considered to be significant.

## Results

**Fig. 2:**
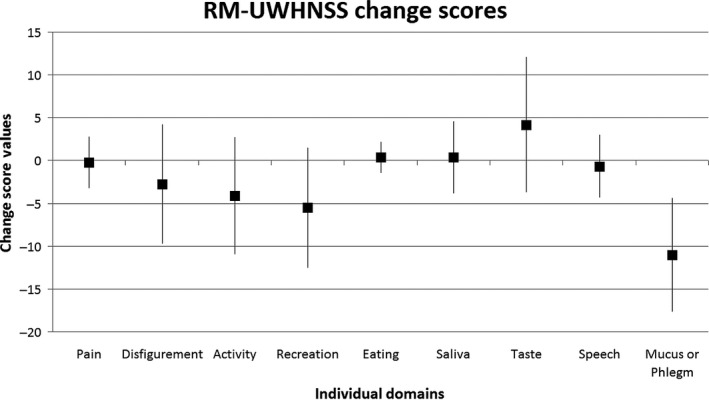
RM‐UWHNSS questionnaire results. Squares represent mean change scores; lines show confidence intervals (95% confidence level). Positive change score means worsening and negative change score means improvement of individual symptom domain. Change scores above 5 points are considered meaningful.

Thirty‐one patients (6 men, 25 women; median age, 52 yr; IQR 42–58 yr) underwent salivary gland scintigraphy before and several months (median, 4.6 months; IQR, 4.3–5.1 months) after thyroid remnant ablation (administered activity, 3.7 GBq). The results are summarised in Table 3. Both in parotid glands and in submandibular glands, the values before and after thyroid remnant ablation were clearly similar, and there was no significant difference either in the uptake (reflecting parenchymal integrity) or in the excretion fraction (reflecting the secretory response to gustatory stimulus). All patients completed the RM‐UWHNSS questionnaire. Only the taste domain mean change score almost reached the +5 point significance level. The mean change scores and their confidence intervals (95% confidence level) are shown in Fig. 2.

**Table 3:** Comparison of ^99m^Tc‐pertechnetate uptake and secretion in salivary glands before and after radioiodine remnant ablation (RRA)

Variable	Before RRA	After RRA	*P* *
Parotid gland			
Uptake (%)	0.14 (0.10–0.20)	0.13 (0.11–0.20)	0.268
Excretion fraction (%)	49.7 (37.1–60.0)	51.4 (42.8–57.5)	0.899
Submandibular gland			
Uptake (%)	0.15 (0.11–0.18)	0.15 (0.11–0.17)	0.855
Excretion fraction (%)	28.3 (21.9–41.1)	35.7 (22.2–42.4)	0.124

Values are median (interquartile range).

* Wilcoxon signed rank test.

Post‐therapeutic scans were performed on all patients. Although the RAI uptake in metallic dental restorations was variable, patients complaining of taste alteration reached higher RAI uptake scores as shown in Table S1.

## Discussion

Our data suggest that a RAI activity of 3.7 GBq, commonly used for thyroid remnant ablation, does not induce important subacute changes in the salivary glands, as assessed by salivary gland scintigraphy. The negative results may be considered reliable, as the calculated power for minimum relevant difference (MIREDIF) of 25% and sample size of 31 ranged between 86% and 96% for the individual variables. Therefore, we are reasonably sure that RAI activity of 3.7 GBq does not cause a decrease in salivary gland functions greater than 25%. Moreover, at least 50% decrease in salivary gland secretion is considered necessary for clinical consequences (33, 34).

Although both salivary gland scintigraphy and sialometry have also been proved to be highly sensitive methods in mild salivary gland impairment in patients with autoimmune diseases (35), sialometric studies prospectively evaluating changes in saliva volume and composition in differentiated thyroid cancer patients are scarce (17). Additionally, studies directly comparing sialometric and scintigraphic changes in patients after thyroid remnant ablation are, to our best knowledge, missing.

The recently published study by klein hesselink *et al*. (17) prospectively comparing total and glandular saliva flow rate and composition revealed a decrease in stimulated whole saliva flow rate (>25%) in 19 patients out of 56 patients undergoing thyroid remnant ablation. However, a decrease of >50%, considered clinically significant by durso (33) and ship *et al.* (34), was observed in only four patients. In addition, most patients (48 out of 56) were treated with 5.55 GBq, and only 6 of the 56 were ablated using the common activity of 3.7 GBq. It can be speculated that with this lower activity, the number of patients with significantly‐decreased saliva flow rate would also be lower, as suggested by some salivary gland scintigraphy studies.

In the report by jeong *et al.* (20), both salivary gland uptake and excretion fraction were evaluated before, and 5 yr after, a single RAI administration (5.55 or 3.7 GBq). For uptake, they used a semi‐quantitative scoring system based on visual assessment of the scintigrams, and for secretion, they calculated the excretion fraction in the same way as done here. Contrary to our design, jeong *et al.* (20) analysed each of the four glands separately, i.e., they did not pool the data from right and left glands. In their sample of 213 patients, they reported a statistically significant decrease in uptake score only in parotid, but not in submandibular glands. A statistically significant decrease in excretion fraction was, however, observed in all glands, except in the right parotid (this laterality was not explained). Nevertheless, these authors reported only a mild RAI‐induced decrease in salivary gland excretion fraction in patients treated with 3.7 GBq (in right parotid glands, a decrease >30% was observed in 12% of patients, while in left parotid glands it was only in 2%, and in submandibular glands a decrease did not occur at all).

However, the reported statistical significance of RAI‐induced injury is still rather contradictory, not only to our results but also to results reported in recent papers by wu *et al*. (21) and, to some extent, by maruoka *et al.* (22) who reported on 194 and 279 patients, respectively. None of them detected a statistically significant decrease in submandibular gland excretion fraction with activities up to 9.5 GBq. Moreover, wu *et al.* (21) did not observe any significant decrease in either function in parotid or submandibular salivary glands of 78 patients treated with activities up to 5.55 GBq. However, a functional damage was detected in 116 patients treated with higher activities, with the parotid gland secretion being the most sensitive variable (21). Similarly, maruoka *et al*. (22) observed a statistically significant decrease in parotid gland uptake and excretion fractions in 193 patients treated with activities ranging from 3.3 to 9.5 GBq (i.e., slightly higher than those used in the present study).

An interesting study examining the relationship of salivary gland functional deterioration with the RAI uptake in salivary glands on post‐therapeutic whole‐body scan was published by jo *et al.* (23) in 2014. The authors analysed, using salivary gland scintigraphy, the changes in salivary gland uptake and excretion in 90 patients before and 5 to 14 months (mean 7.4) after thyroid remnant ablation. The administered RAI activities ranged from 3.7 to 9.3 GBq (3.7, 5.6, 7.4 and 9.3 GBq in 38, 46, 3 and 3 patients, respectively). In the whole group, a significant decrease in parotid gland uptake and excretion and (to a lesser extent) reduced submandibular gland excretion was observed. Regrettably, the subgroups were not analysed separately, and therefore we cannot easily compare their data with our patients treated with the activity of 3.7 GBq. Moreover, the extent of parotid gland deterioration was significantly more pronounced in patients with RAI retention in parotid glands as revealed by post‐therapeutic scan. Interestingly, in our post‐therapeutic scans, performed in exactly the same setting, there was no RAI uptake in salivary glands following the activity of 3.7 GBq (23).

Contrary to all the aforementioned papers, upadhyaya *et al*. (24) observed an increased uptake and stable excretion fraction in all four main salivary glands in 36 patients 6 month after RAI administration (activity of 3.7 GBq). The authors explained the increased uptake by a rather elusive ‘compensatory mechanism to maintain the basic stability of the secretory function’. In their study protocol, however, there was an important difference from all the other studies, including ours. They also performed the follow‐up salivary gland scintigraphy under thyroid‐ and hormone‐stimulating conditions (either during a diagnostic RAI scan or another RAI therapy), which might have increased the uptake via NIS activation.

Interestingly, there might be another confounding factor in early (within hours) use of a sialagogue after RAI administration. In all the aforementioned studies (excluding studies by maruoka *et al*. (22) and upadhyaya *et al*. (24)), the patients were encouraged to use vitamin C, sour candies, and the like immediately after the RAI administration, in an attempt to decrease salivary gland injury. However, doing so may have the opposite effect. Careful dosimetric studies by jentzen *et al.* (28, 29) have clearly demonstrated higher absorbed doses to salivary glands following their early stimulation. We did not use sialagogues, which might have made our patients less sensitive to salivary gland damage. In the study by wu *et al.* (21) with similar results, sialagogues were used but also prednisone (15 mg, twice daily) was administered, which might have attenuated the radiation injury.

The only symptom concerning our patients was a mild taste alteration, which did not necessarily result from salivary gland damage — as suggested by scintigraphic results and the absence of subjective worsening of salivation. This apparent discrepancy may be explained by an intensive and prolonged chemical binding of RAI to the dental amalgam (36), clearly visible in Fig. S1 and pronounced in patients with taste change complaints (Table S1). Considering that the tongue is in close contact with radioactive metallic dental restorations, we can hypothesize that taste is more likely to be altered by a longer lasting irradiation of the taste buds and small lingual salivary glands than by a major salivary gland radiation injury (37, 38).

An important issue to address is multiple RAI administrations. Salivary flow rates (stimulated and unstimulated), sialochemical analyses of both stimulated and unstimulated saliva, and xerostomia‐related complaints were evaluated in a recently published cross‐sectional study by selvakumar *et al*. (39) on 63 long‐term survivors of pediatric differentiated thyroid cancer treated with median activity of 5.88 GBq (IQR 2.92–12.95 GBq). Patients treated with multiple RAI administrations (31 out of 63) had significantly lower amylase levels in stimulated saliva as well as significantly more pronounced xerostomia‐related complaints. The authors also observed activity‐dependent decrease in stimulated saliva flow rates and total protein and amylase levels, which were significantly lower in those treated with activities ≥7.4 GBq. Xerostomia‐related complaints were significantly more pronounced even in patients treated by activities ≥3.7 GBq. This is of great importance considering that thyroid remnant ablation with insufficient RAI activity may lead to repeated RAI administration with cumulative activity above 3.7 GBq (40, 41).

Based on our data and comparison with other studies, it may be concluded that thyroid remnant ablation with the usual activity of 3.7 GBq, without salivary gland stimulation (sialagogue administration) after RAI administration, does not deteriorate salivary gland uptake and secretory functions. Therefore, the concerns about a putative salivary gland radiation injury and consequent oral health deterioration after a single administration of 3.7 GBq seem to be unjustified. In addition, the traditionally used early sialagogues may be counterproductive and should be avoided.

## Conflicts of interest

The authors declare no conflicts of interest.

## Supporting information


**Figure S1** **.** RAI uptake on the surface of metallic dental restorations detected by ^131^I post‐therapeutic Single Photon Emission Tomography/Low Dose CT (SPECT/CT)
**Table S1** **.** Comparison of RAI uptake in dental restorations in all patients and those with and without taste alterations

## References

[1] Davies L , Welch HG . Increasing incidence of thyroid cancer in the United States, 1973–2002. JAMA 2006; 295: 2164–2167. 16684987 10.1001/jama.295.18.2164

[2] Lukas J , Drabek J , Lukas D , Dusek L , Gatek J . The epidemiology of thyroid cancer in the Czech Republic in comparison with other countries. Biomed Pap Med Fac Univ Palacky Olomouc Czech Repub 2013; 157: 266–275. 23132514 10.5507/bp.2012.086

[3] Mazzaferri EL , Jhiang SM . Long‐term impact of initial surgical and medical therapy on papillary and follicular thyroid cancer. Am J Med 1994; 97: 418–428. 7977430 10.1016/0002-9343(94)90321-2

[4] Haugen BR , Alexander EK , Bible KC , Doherty GM , Mandel SJ , Nikiforov YE , Pacini F , Randolph GW , Sawka AM , Schlumberger M , Schuff KG , Sherman SI , Sosa JA , Steward DL , Tuttle RM , Wartofsky L . 2015 American thyroid association management guidelines for adult patients with thyroid nodules and differentiated thyroid cancer: the American thyroid association guidelines task force on thyroid nodules and differentiated thyroid cancer. Thyroid 2016; 26: 1–133. 26462967 10.1089/thy.2015.0020 PMC4739132

[5] Mandel SJ , Mandel L . Radioactive iodine and the salivary glands. Thyroid 2000; 13: 265–271. 10.1089/105072503321582060 12729475

[6] Tavares C , Coelho MJ , Eloy C , Melo M , da Rocha AG , Pestana A , Batista R , Ferreira LB , Rios E , Selmi‐Ruby S , Cavadas B , Pereira L , Sobrinho Simões M , Soares P . NIS expression in thyroid tumors, relation with prognosis clinicopathological and molecular features. Endocr Connect 2018; 7: 78–90. 29298843 10.1530/EC-17-0302 PMC5754505

[7] Dohán O , De la Vieja A , Paroder V , Riedel C , Artani M , Reed M , Ginter CS , Carrasco N . The sodium/iodide Symporter (NIS): characterization, regulation, and medical significance. Endocr Rev 2003; 24: 48–77. 12588808 10.1210/er.2001-0029

[8] Pink R , Simek J , Vondrakova J , Faber E , Michl P , Pazdera J , Indrak K . Saliva as a diagnostic medium. Biomed Pap 2009; 153: 103–110. 10.5507/bp.2009.017 19771133

[9] Twetman S . Prevention of dental caries as a non‐communicable disease. Eur J Oral Sci 2018; 126(Suppl): 19S–25S. 10.1111/eos.12528 30178558

[10] Alexander C , Bader JB , Schaefer A , Finke C , Kirsch CM . Intermediate and long‐term side effects of high‐dose radioiodine therapy for thyroid carcinoma. J Nucl Med 1998; 39: 1551–1554. 9744341

[11] Walter MA , Turtschi CP , Schindler C , Minnig P , Müller‐Brand J , Müller B . The dental safety profile of high‐dose radioiodine therapy for thyroid cancer: long‐term results of a longitudinal cohort study. J Nucl Med 2007; 48: 1620–1625. 17873131 10.2967/jnumed.107.042192

[12] Grewal RK, Larson SM , Pentlow CE , Pentlow KS , Gonen M , Qualey R , Natbony L , Tuttle RM . Salivary gland side effects commonly develop several weeks after initial radioactive iodine ablation. J Nucl Med 2009; 50: 1605–1610. 19759114 10.2967/jnumed.108.061382

[13] Hollingsworth B , Senter L , Zhang X , Brock GN , Jarjour W , Nagy R , Brock P , Coombes KR , Kloos RT , Ringel MD , Sipos J , Lattimer I , Carrau R , Jhiang SM . Risk factors of (131)^I^‐induced salivary gland damage in thyroid cancer patients. J Clin Endocrinol Metab 2016; 101: 4085–4093. 27533304 10.1210/jc.2016-1605 PMC5095242

[14] Blumhardt R , Wolin EA , Phillips WT , Salman UA , Walker RC , Stack Jr BC , Metter D . Current controversies in the initial post‐surgical radioactive iodine therapy for thyroid cancer: a narrative review. Endocr Relat Cancer 2014; 21: R473–484. 25277792 10.1530/ERC-14-0286

[15] Jentzen W , Hobbs RF , Stahl A , Knust J , Sgouros G , Bockisch A . Pre‐therapeutic (124)I PET(/CT) dosimetry confirms low average absorbed doses per administered (131)I activity to the salivary glands in radioiodine therapy of differentiated thyroid cancer. Eur J Nucl Med Mol Imaging 2010; 37: 884–895. 20069293 10.1007/s00259-009-1351-2 PMC2854857

[16] Klutmann S , Bohuslavizki KH , Kröger S , Bleckmann C , Brenner W , Mester J , Clausen M . Quantitative salivary gland scintigraphy. J Nucl Med Technol 1999; 27: 20–26. 10322570

[17] Klein Hesselink EN , Brouwers AH , de Jong JR , van der Horst‐Schrivers AN , Coppes RP , Lefrandt JD , Jager PL , Vissink A , Links TP . Effects of radioiodine treatment on salivary gland function in patients with differentiated thyroidcarcinoma: a prospective study. J Nucl Med 2016; 57: 1685–1691. 27339871 10.2967/jnumed.115.169888

[18] Fogelman I , Clarke SEM , Cook G , Gnanasegaran G . An atlas of clinical nuclear medicine. Boca Raton, FL: CRC Press, 2014; 1245–1256.

[19] Helman J , Turner RJ , Fox PC , Baum BJ . 99mTc‐pertechnetate uptake in parotid acinar cells by the Na+/K+/Cl‐ co‐transport system. J Clin Invest 1987; 79: 1310–1313. 3033020 10.1172/JCI112954 PMC424370

[20] Jeong SY , Kim HW , Lee SW , Ahn BC , Lee J . Salivary gland function 5 years after radioactive iodine ablation in patients with differentiated thyroid cancer: direct comparison of pre‐ and post‐ablation scintigraphies and their relation to xerostomia symptoms. Thyroid 2013; 23: 609–616. 23153322 10.1089/thy.2012.0106 PMC3643252

[21] Wu JQ , Feng HJ , Ouyang W , Sun YG , Chen P , Wang J , Xian JL , Huang LH . Systematic evaluation of salivary gland damage following I‐131 therapy in differentiated thyroid cancer patients by quantitative scintigraphy and clinical follow‐up. Nucl Med Commun 2015; 36: 819–826. 25932534 10.1097/MNM.0000000000000325

[22] Maruoka Y , Baba S , Isoda T , Kitamura Y , Abe K , Sasaki M , Honda H . A functional scoring system based on salivary gland scintigraphy for evaluating salivary gland dysfunction secondary to (131)I therapy in patients with differentiated thyroid carcinoma. J Clin Diagn Res 2017; 11: TC23–TC28. 28969240 10.7860/JCDR/2017/27340.10431 PMC5620881

[23] Jo KS , An YS , Lee SJ , Soh EY , Lee J , Chung YS . Significance of salivary gland radioiodine retention on post‐ablation (131)I scintigraphy as a predictor of salivary gland dysfunction in patients with differentiated thyroid carcinoma. Nucl Med Mol Imaging 2014; 48: 203–211. 25177377 10.1007/s13139-014-0274-4 PMC4145088

[24] Upadhyaya A , Meng Z , Wang P , Zhang G , Jia Q , Tan J , Li X , Hu T , Liu N , Zhou P , Wang S , Liu X , Wang H , Zhang C , Zhao F , Yan Z . Effects of first radioiodine ablation on functions of salivary glands in patients with differentiated thyroid cancer. Medicine (Baltimore) 2017; 96: e7164. 28640094 10.1097/MD.0000000000007164 PMC5484202

[25] Schlumberger M , Catargi B , Borget I , Deandreis D , Zerdoud S , Bridji B , Bardet S , Leenhardt L , Bastie D , Schvartz C , Vera P , Morel O , Benisvy D , Bournaud C , Bonichon F , Dejax C , Toubert ME , Leboulleux S , Ricard M , Benhamou E . Tumeurs de la Thyroïde Refractaires Network for the Essai Stimulation Ablation Equivalence Trial . Strategies of radioiodine ablation in patients with low‐risk thyroid cancer. N Engl J Med 2012; 366: 1663–1673. 22551127 10.1056/NEJMoa1108586

[26] Mallick U , Harmer C , Yap B , Wadsley J , Clarke S , Moss L , Nicol A , Clark PM , Farnell K , McCready R , Smellie J , Franklyn JA , John R , Nutting CM , Newbold K , Lemon C , Gerrard G , Abdel‐Hamid A , Hardman J , Macias E , Roques T , Whitaker S , Vijayan R , Alvarez P , Beare S , Forsyth S , Kadalayil L , Hackshaw A . Ablation with low‐dose radioiodine and thyrotropin alfa in thyroid cancer. N Engl J Med 2012; 366: 1674–1685. 22551128 10.1056/NEJMoa1109589

[27] Wyatt G , Pugh SL , Wong RK , Sagar S , Singh AK , Koyfman SA , Nguyen‐Tân PF , Yom SS , Cardinale FS , Sultanem K , Hodson I , Krempl GA , Lukaszczyk B , Yeh AM , Berk L . Xerostomia health‐related quality of life: NRG oncology RTOG 0537. Qual Life Res 2016; 25: 2323–2333. 26914104 10.1007/s11136-016-1255-0 PMC4982852

[28] Nakada K , Ishibashi T , Takei T , Hirata K , Shinohara K , Katoh S , Zhao S , Tamaki N , Noguchi Y , Noguchi S . Does lemon candy decrease salivary gland damage after radioiodine therapy for thyroid cancer? J Nucl Med 2005; 46: 261–266. 15695785

[29] Jentzen W , Balschuweit D , Schmitz J , Freudenberg L , Eising E , Hilbel T , Bockisch A , Stahl A . The influence of saliva flow stimulation on the absorbed radiation dose to the salivary glands during radioiodine therapy of thyroid cancer using 124I PET(/CT) imaging. Eur J Nucl Med Mol Imaging 2010; 37: 2298–2306. 20625723 10.1007/s00259-010-1532-z

[30] Jentzen W , Richter M , Nagarajah J , Poeppel TD , Brandau W , Dawes C , Bockisch A , Binse I . Chewing‐gum stimulation did not reduce the absorbed dose to salivary glands during radioiodine treatment of thyroid cancer as inferred from pre‐therapy (124)I PET/CT imaging. EJNMMI Physics 2014; 1: 100. 26501458 10.1186/s40658-014-0100-1 PMC4545453

[31] Vissink A , Mitchell JB , Baum BJ , Limesand KH , Jensen SB , Fox PC , Elting LS , Langendijk JA , Coppes RP , Reyland ME . Clinical management of salivary gland hypofunction and xerostomia in head‐and‐neck cancer patients: successes and barriers. Int J Radiat Oncol Biol Phys 2010; 78: 983–991. 20970030 10.1016/j.ijrobp.2010.06.052 PMC2964345

[32] Hoffman KE , Pugh SL , James JL , Scarantino C , Movsas B , Valicenti RK , Fortin A , Pollock J , Kim H , Brachman DG , Berk LB , Bruner DW , Kachnic LA . The impact of concurrent granulocyte‐macrophage colony‐stimulating factor on quality of life in head and neck cancer patients: results of the randomized, placebo‐controlled Radiation Therapy Oncology Group 9901 trial. Qual Life Res 2014; 23: 1841–1858. 24492945 10.1007/s11136-014-0628-5 PMC4533105

[33] Durso SC . Oral manifestations of disease In: Kasper DL, Fauci AS, Longo DL, Braunwald E, Hauser SL, Jameson JL, eds. Harrison´s principles of internal medicine. New York, NY: Mc Graw‐Hill, 2005; 194–201.

[34] Ship JA , Fox PC , Baum BJ . How much saliva is enough? 'Normal' function defined. J Am Dent Assoc 1991; 122: 63–69. 10.14219/jada.archive.1991.0098 2019691

[35] Dugonjić S , Stefanović D , Ethurović B , Spasić‐Jokić V , Ajdinović B . Evaluation of diagnostic parameters from parotid and submandibular dynamic salivary glands scintigraphy and unstimulated sialometry in Sjögren's syndrome. Hell J Nucl Med 2014; 17: 116–122. 25097897

[36] Burlison JS , Hartshorne MF , Voda AM , Cocks FH , Fair JR . SPECT/CT localization of oral radioiodine activity: a retrospective study and in‐vitro assessment. Nucl Med Commun 2013; 34: 1216–1222. 24128897 10.1097/MNM.0000000000000004 PMC3815121

[37] Deshpande TS , Blanchard P , Wang L , Foote RL , Zhang X , Frank SJ . Radiation‐related alterations of taste function in patients with head and neck cancer: a systematic review. Curr Treat Options Oncol 2018; 19: 72. 30411162 10.1007/s11864-018-0580-7 PMC6244914

[38] Dietlein M , Drzezga A . Taste dysfunction (dysgeusia) and radioiodine therapy of thyroid cancer – be aware of side effects by antidepressants and sedatives. Nuklearmedizin 2017; 56: 125–131. 28715040 10.3413/Nukmed-0906-17-06

[39] Selvakumar T , Nies M , Klein Hesselink MS , Brouwers AH , van der Horst‐Schrivers ANA , Klein Hesselink EN , Tissing WJE , Vissink A , Links TP , Bocca G , Burgerhof JGM , van Dam EWCM , Havekes B , van den Heuvel‐Eibrink MM , Corssmit EPM , Kremer LCM , Netea‐Maier RT , van der Pal HJH , Peeters RP , Smit JWA , Plukker JTM , Ronckers CM , van Santen HM . Long‐term effects of radioiodine treatment on salivary gland function in adult survivors of pediatric differentiated thyroid carcinoma. J Nucl Med. 2019; 60: 172–177. 10.2967/jnumed.118.212449 30504138

[40] Verburg FA , Mäder U , Reiners C , Hänscheid H . Long‐term survival in differentiated thyroid cancer is worse after low‐activity initial post‐surgical 131I therapy in both high‐ and low‐risk patients. J Clin Endocrinol Metab 2014; 99: 4487–4496. 25259907 10.1210/jc.2014-1631

[41] Kukulska A , Krajewska J , Gawkowska‐Suwińska M , Puch Z , Paliczka‐Cieslik E , Roskosz J , Handkiewicz‐Junak D , Jarzab M , Gubała E , Jarzab B . Radioiodine thyroid remnant ablation in patients with differentiated thyroid carcinoma (DTC): prospective comparison of long‐term outcomes of treatment with 30, 60 and 100 mCi. Thyroid Res 2010; 3: 9. 21040579 10.1186/1756-6614-3-9 PMC2989933

