# Supplementary material for: Scintigraphic evaluation of salivary gland function in thyroid cancer patients after radioiodine remnant ablation
Source: Eur J Oral Sci. 2020 Apr 2;128(3):204–10. doi: 10.1111/eos.12689 (PMC7318700; doi:10.1111/eos.12689)
Supplement: Supplementary file 1 — Figure S1 . RAI uptake on the surface of metallic dental restorations detected by 131I post‐therapeutic Single Photon Emission Tomography/Low Dose CT (SPECT/CT) Table S1 . Comparison of RAI uptake in dental restorations in all patients and those with and without taste alterations [file EOS-128-204-s001.pdf]

SUPPORTING INFORMATION

Scintigraphic evaluation of salivary gland function in thyroid cancer patients after radioiodine remnant ablation

KRČÁLOVÁ E, HORÁČEK J, GABALEC F, ŽÁK P, DOLEŽAL J

University Hospital Hradec Králové, Hradec Králové, Czech Republic
Charles University, Hradec Králové, Czech Republic

Fig. S1. RAI uptake on the surface of metallic dental restorations detected by ^{131}I post-therapeutic Single Photon Emission Tomography/Low Dose CT (SPECT/CT). CT images (a) depict mandible with metallic fillings, SPECT images (b) shows RAI uptake and SPECT/CT fused images (c) precisely locate RAI uptake into metallic fillings.

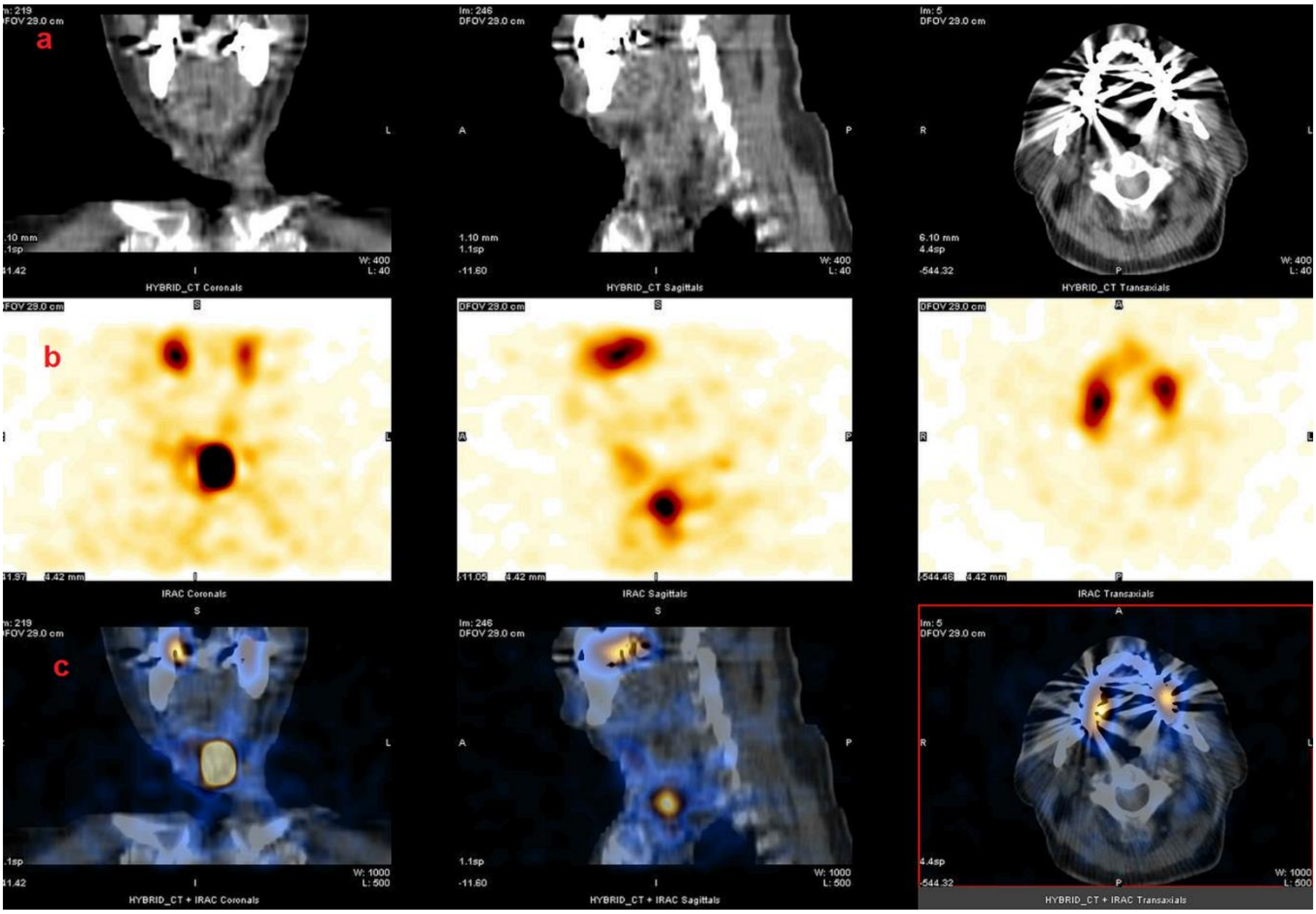

Table S1 Comparison of RAI uptake in dental restorations in all patients and those with and without taste alterations

Variable	All patients (n=6)	Asymptomatic patients (n=25)	Symptomatic patients (n=6)
RUS	2.0 (0.0-3.0)	1.0 (0.0-2.0)	4.0 (3.3-4.0)

RUS radioiodine uptake score, values are median (interquartile range)
